# EGb 761 reduces Ca^2+^ influx and apoptosis after pentylenetetrazole treatment in a neuroblastoma cell line

**DOI:** 10.3389/fncel.2023.1195303

**Published:** 2023-09-07

**Authors:** Ishak Suat Ovey, Ahmet Ozsimsek, Halil Aziz Velioglu, Ozlem Altay, Adil Mardinoglu, Burak Yulug

**Affiliations:** ^1^Department of Physiology, Faculty of Medicine, Alanya Alaaddin Keykubat University, Antalya, Türkiye; ^2^Department of Neurology and Neuroscience, Faculty of Medicine, Alanya Alaaddin Keykubat University, Antalya, Türkiye; ^3^Department of Neuroscience, Faculty of Medicine, Istanbul Medipol University, Istanbul, Türkiye; ^4^Center for Psychiatric Neuroscience, Feinstein Institutes for Medical Research, Manhasset, NY, United States; ^5^KTH Royal Institute of Technology, Stockholm, Sweden

**Keywords:** transient receptor potential (TRP) channels, EGb 761, epilepsy EGb, neuroprotección, ginseng

## Abstract

**Background:**

Transient receptor potential (TRP) channels have been found to have significant implications in neuronal outgrowth, survival, inflammatory neurogenic pain, and various epileptogenic processes. Moreover, there is a growing body of evidence indicating that transient receptor potential (TRP) channels have a significant impact on epilepsy and its drug-resistant subtypes.

**Objective:**

We postulated that *E*Gb 761 would modulate TRPA1 channels, thereby exhibiting anti-inflammatory and neuroprotective effects in a neuroblastoma cell line. Our rationale was to investigate the impact of *EGb 761* in a controlled model of pentylenetetrazole-induced generalized epilepsy.

**Methodology:**

We evaluated the neuroprotective, antioxidant and anti-apoptotic effects of *E*Gb 761 both before and after the pentylenetetrazole application in a neuroblastoma cell line. Specifically, we focused on the effects of EGB 761 on the activity of Transient receptor potential (TRP) channels.

**Results:**

EGb 761 applications both before and after the pentylenetetrazole incubation period reduced Ca release and restored apoptosis, ROS changes, mitochondrial depolarization and caspase levels, suggesting a prominent prophylactic and therapeutic effect of *E*Gb 761 in the pentylenetetrazole-induced epileptogenesis process.

**Conclusion:**

Our basic mechanistic framework for elucidating the pathophysiological significance of fundamental ion mechanisms in a pentylenetetrazole treated neuroblastoma cell line provided compelling evidence for the favorable efficacy and safety profile of Egb 761 in human-relevant *in vitro* model of epilepsy. To the best of our knowledge, this is the first study to investigate the combined effects of EGb 761 and pentylenetetrazole on TRP channels and measure their activation level in a relevant model of human epileptic diseases.

## Graphical Abstract

The underlying protective mechanisms of GB. 
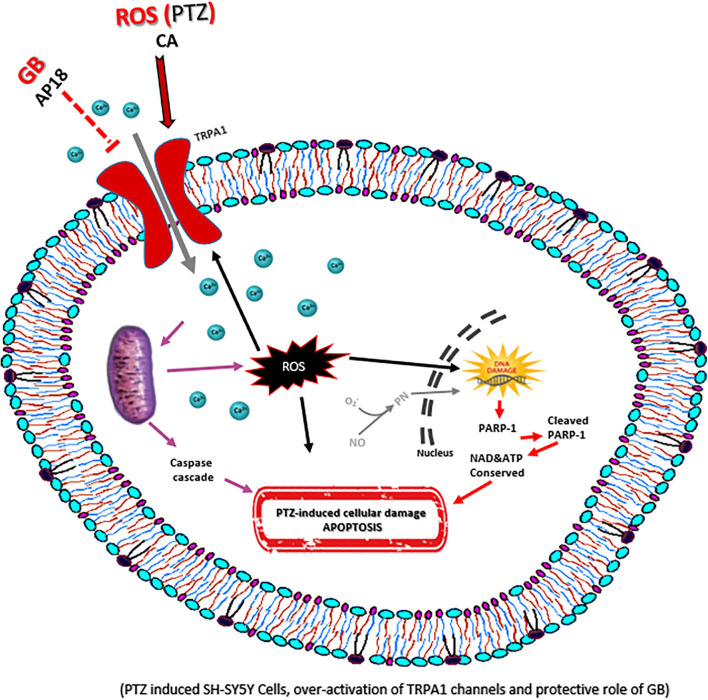

## Introduction

*EGb 761* has been used therapeutically and experimentally to treat a range of medical conditions (von Boetticher, 2011; Gauthier and Schlaefke, 2014; von Gunten et al., 2016; Savaskan et al., 2018; Hort et al., 2023). Several preclinical and clinical research suggests that *EGb 761* may exhibit significant neuroprotective and even anticonvulsant efficacy (Chung et al., 2006; Ilhan et al., 2006; Zhang et al., 2015).

Transient receptor potential (TRP) channels not only play an important role in neuronal outgrowth, survival, and inflammatory neurogenic pain, but are also believed to play a pivotal role in various epileptogenic processes, including a number of critical secondary processes, such as cell death and neuroinflammation (Caterina and Julius, 2001; Julius and Basbaum, 2001). Hunt et al. (2012) for example, established that TRPA channels may be important for controlling glutamate toxicity and mitochondrial depolarization, both of which may result in a decreased epileptic threshold, eventually causing neuronal cell death (Nazıroğlu, 2009). Subsequent research also revealed that TRP channel expression causes hyperexcitability and epileptiform activity in animals (Iannotti et al., 2014). Consistent with this, knockout animal experiments targeting particular TRP channels have shown considerably reduced epileptic activity, as well as decreased seizure-induced neuronal cell death in the hippocampus (Phelan et al., 2013). TRPVC has also been implicated in the pathogenesis of epilepsy in human research. Some TRPM2 channels, for example, are co-expressed with the EF-hand domain-containing protein 1 gene, which has been linked to an elevated risk of juvenile myoclonic epilepsy (Katano et al., 2012). TRPM7 has also been demonstrated to be active during epilepsy (Aarts and Tymianski, 2005).

TRPV1 channels are expressed similarly throughout brain regions important for epilepsy pathogenesis, such as the cerebral cortex, hippocampus, and amygdala (Cristino et al., 2006; Leistner and Drewke, 2010; Nazıroglu, 2015; Naziroğlu and Övey, 2015). TRPV1 expression is enhanced in the hippocampus of rats and the dentate gyrus of mice with temporal lobe epilepsy, as well as in the brains of individuals with temporal lobe epilepsy (Bhaskaran and Smith, 2010; Sun et al., 2013; Saffarzadeh et al., 2015). In a similar vein, TRPA1 plays a critical role in several neurological diseases, including also the epilepsy (Günaydın et al., 2020). These results are consistent with the fact that blocking the TRPA1 channel prevents seizures and epilepsy induced Ca^2+^ entry in the hippocampus (Naziroğlu and Övey, 2015). As previously discovered for other TRP channels, several human studies also suggested that TrpC1 increases in cortical lesions of epilepsy patients (Zang et al., 2015). Based on these results, the question of how modification of the TRPM channel may alter epileptogenesis and the associated interactive processes described above remains an intriguing one. These results suggest that epilepsy should be considered not just as a simple clinical seizure symptom, but also as a comprehensive process with all its causes and potential effects on neuronal cell death and neuroinflammation (Yulug et al., 2018). A number of studies have shown that epilepsy may cause neuronal cell death due to mitochondrial instability, caspase release, and glutamate toxicity. Various human investigations have revealed that epilepsy is also associated with important cognitive and neuropsychiatric disorders such epileptic personality, cognitive decline, and even dementia (Devinsky and Vazquez, 1993; Haut et al., 2009). Given these results, we postulated that *EGb 761* would exhibit anti-inflammatory, and neuroprotective properties in neuroblastoma cell lines. We thus investigated *EGb 761* and examined its neuroprotective properties by modulating TRPV1 channels. The purpose of this research was to examine the impact of *EGb 761* on PTZ-induced seizures, as well as alterations in brain glutamate, caspase, and antioxidant defenses based on the well-known convulsive properties of Pentylenetetrazole (PTZ) which is a GABA-A receptor antagonist. Herein several studies have shown that intraperitoneal injection of PTZ into an animal might induce a dose-dependent effect ranging from a sub-convulsive chemical kindling to direct convulsive effect in *in vivo* and *in vitro* models of epilepsy (Shimada and Yamagata, 2018). Epilepsy modeling in experimental animals and cell culture media is generally performed using pentylenetetrazole (PTZ). In the present study, we aimed to compare the protective and therapeutic role of ginkgo bloba in PTZ-treated cells and to determine the role of TRPA1 channels in this interaction. For this purpose, although our study was logically planned as four main groups as Control, Gingko bloba + PTZ, PTZ and PTZ + Gingko bloba; it was planned as a total of seven groups using channel inhibitors and stimulators together with the control group. There are two main objectives in our study, the first one is to determine which of the protective and therapeutic effects of Gingko bloba is more effective in PTZ treated cells, and the second one is to determine the role of TRPPA1 channels activated by oxidative stress in this interaction. Therefore, the difference between the stimulator and inhibitor + stimulator groups in the same chemical treated groups shows the channel function (effect).

Considering the notable occurrence of drug-resistant forms of epilepsy and the role of TRPM channels in the pathogenesis of epilepsy and its drug-resistant variants, which can lead to cognitive decline and dementia, this study has a unique/distinct objective of assessing the potential anti-convulsant and neuroprotective properties of EGB 761 by a human-relevant *in vitro* epilepsy model that simulates the drug-resistant epilepsy process mediated by TRCP channels observed in humans.

## Materials and methods

### Cell culture

A human neuroblastoma cell line (SH-SY5Y) was obtained from the American Type Culture Collection (ATCC) (Manassas, VA, USA). Cells were cultured in Ham’s F12 and Dulbecco’s modified eagle media at a 1:1 ratio containing 10% fetal bovine serum (FBS) (Fisher Scientific) and a 1% pen./strep. antibiotic combination in 8–10 flasks (filter cap, sterile, 5 ml, 25 cm^2^). Cells were incubated in T25 flasks at 37°C at 5% CO_2_ in a humidified incubator. Once the cells reached 75–85% confluence, they were incubated with the chemical compounds described in the section describing the study groups below. Cells were examined daily for evidence of contamination. After chemical treatments, washed cells were detached with 0.25% Trypsin–EDTA from T25 flasks, after which 4 ml fresh medium was added per flask. Cell suspensions were collected using a recharged automatic pipette and transferred into 15 ml Falcon tubes. Cells were centrifuged (100 × *g*, 5 min), after which the resulting supernatants were removed. Centrifugation was repeated by adding fresh medium to the sterile Falcon tubes to wash the cells and make them ready for use in the experiments. Initially, 500,000 cells were seeded in flasks and checked daily. The cells reached 75% confluence on average on the 3rd day after seeding and were then treated with the chemicals mentioned in the study protocol for the specified times. Ten wells for each group were analyzed in a 96-well plate with 50,000 cells per well in a fully automatic injector plate reader. After treatment with chemicals, programmed cell death (apoptosis) assay was performed separately for each group. The differences between the groups are also presented in Figure 2 (apoptosis results).

### Reagents/dyes/chemicals

The APOPercentage assay and releasing buffer were purchased from Biocolor (Belfast, Northern Ireland, UK). Dihydrorhodamine-123 (DHR 123) and pentylenetetrazol were obtained from Sigma Aldrich (St. Louis, MO, USA), and Caspase-3 (AC-DEVD-AMC) and Caspase-9 (AC-LEHD-AMC) substrates from Enzo (Lausen, Switzerland). Fura 2 (AM) calcium florescent dye was purchased from Calbiochem (Darmstadt, Germany) and Pluronic^®^ F-127 from Biovision (San Francisco, CA, USA). A mitochondrial stain 5,50, 6,60-tetrachloro-1,10,3,30- tetraethylbenzimidazolyl carbocyanine iodide (JC-1) and Probenecid were purchased from Santa Cruz (Dallas, TX, USA). *EGb 761* was provided by AbdIbr Pharmaceuticals.

### Groups

The study was planned to include seven main groups as described below:

**Group 1 (Control):** none of the study drugs were used, and SH-SY5Y was preserved in a flask with the same cell culture conditions.

**Group 2 (*****EGb 761*** **+ PTZ):** cells in this group were incubated with 25 μg/ml *EGb 761* for 24 h and then incubated with 30 mM pentylenetetrazole for 24 h (di Meo et al., 2020; Taskiran and Ergul, 2021).

**Group 3 (*****EGb 761*** **+ PTZ + AP18):** cells in this group were incubated with 25 μg/ml *EGb 761* for 24 h and then incubated with 30 mM pentylenetetrazole for 24 h and finally incubated with TRPA1 channel antagonist AP18 (0.1 mM, 30 min).

**Group 4 (PTZ):** cells in this group were incubated with 30 mM pentylenetetrazole for 24 h (Taskiran and Ergul, 2021).

**Group 5 (PTZ + AP18):** cells in this group were incubated with 30 mM pentylenetetrazole for 24 h and then incubated with TRPA1 channel antagonist AP18 (0.1 mM, 30 min).

**Group 6 (PTZ +** ***EGb 761*****):** cells in this group were incubated with 30 mM pentylenetetrazole for 24 h and then incubated with 25 μg/ml *EGb 761* leaf extract for 24 h (di Meo et al., 2020; Taskiran and Ergul, 2021).

**Group 7 (PTZ +** ***EGb 761*** **+ AP18):** cells in this group were incubated with 30 mM pentylenetetrazole for 24 h and then incubated with 25 μg/ml *EGb 761* for 24 h and finally incubated with TRPA1 channel antagonist AP18 (0.1 mM, 30 min).

During calcium signaling analysis (Fura-2/AM), cells were stimulated on the 20th cycles with 0.1 mM cinnamaldehyde (CA) in the presence of 1.2 mM calcium and calcium-free buffer in the extracellular environment. For apoptosis, intracellular reactive oxygene species, mitochondrial depolarization, Caspase-3, and Caspase-9 experiments, the cells were further treated with TRPA1 channel agonist CA (0.1 mM, 10 min) for activation of the TRPA1 channel before the relevant analysis.

### Measurements of intracellular calcium and Fura-2 loading

Fura 2 AM (acetoxymethyl ester) dye was used to measure intracellular calcium levels in SH-SY5Y cells. The cells were incubated with 1.2 mM CaCl_2_ and calcium-free HEPES-buffered saline [HBS; 5 mM KCl, 145 mM NaCl, 10 mM D-glucose, 1 mM MgCl_2_, 10 mM HEPES and 0.1% (w/v) bovine serum albumin (BSA); pH 7.4] containing 5 μM Fura-2 AM and 0.05% (w/v) Pluronic F-127 for 60 min at 37°C and in the same solution in the dark. The loaded cells were washed twice with HBS and covered with 1 ml of HBS supplemented with 2.5 mM probenecid for at least 20 min at 37°C in the dark to allow for Fura-2 AM de-esterification. Fluorescence intensity at 510 nm (emission) was determined in individual wells using a plate reader equipped with an automated injection system (SynergyTM H1, Biotek, USA) at alternating excitation wavelengths of 340 and 380 nm every 3 s for 50 acquisition cycles. During the measurement of intracellular calcium signaling, TRPA1 channels were stimulated by means of an automatic injector with CA (0.1 mM) on 20th cycle. Measurement of (Ca^2+^) including staining process modification was performed following the method described by Martinez et al. (2016) and Övey and Güler (2020).

### Apoptosis assay

APOPercentageTM (the cell apoptosis assay) was used for the detection and quantification of apoptosis. The APOPercentage dye is actively bound to phosphatidylserine lipids and transferred into the cells, and apoptotic cells are stained red. The apoptosis analysis procedure was performed according to the manufacturer’s instructions and as described by Öz and Çelik (2016) and Övey and Güler (2020). The SH-SY5Y cells were analyzed for apoptotic cell detection using spectrophotometry (multiplate reader) at 550 nm (SynergyTM H1, Biotek, USA), and the results were shown as -fold changes over the pre-treatment level (experiment/control).

### Intracellular ROS production measurement and caspase 3–9 activity assays

Dihydrorhodamine-123 (DHR-123) passes easily through the cell membrane. Inside the SH-SY5Y cells, DHR-123 is oxidized to cationic rhodamine-123 (Rh-123) which is localized in the mitochondria and exhibits green fluorescence. The cells (106 cells/ml for per group) were incubated with 20 μm DHR-123 as florescent oxidant dye at 37°C for 25 min (Öz and Çelik, 2016). A SynergyTM H1 automatic microplate reader device was used for determining Rh-123 fluorescent intensities. Analyses were performed at a 488 nm excitation wavelength and 543 nm emission wavelength. We presented the data as -fold changes over the pre-treatment level.

Caspase 3 (AC-DEVD-AMC) and Caspase 9 (AC-LEHD-AMC) substrate cleavages were measured with a SynergyTM H1 microplate reader (Biotek, USA) at 360 nm and 460 nm wavelengths (excitation/emission, respectively). Caspase 3 and Caspase 9 activity evaluation methods were based on previous descriptions (Öz and Çelik, 2016). The values were expressed as fluorescent units/mg protein and shown as -fold changes from pre-treatment levels (experimental/control, respectively).

### Mitochondrial membrane potential (JC-1) analyses

Mitochondrial membrane potential fluorescence dye [JC1(1 μM)] intensity was evaluated at a 485 nm (green) excitation wavelength and an emission wavelength of 535 nm, with the red signal at 540 nm excitation and a 590 nm emission wavelengths (SynergyTM H1, Biotek, USA) (Öz and Çelik, 2016; Övey and Güler, 2020). Data are presented as emission ratios (590/535). Mitochondrial membrane potential changes were quantified as the integral of the decrease in the JC1 fluorescence experimental/control ratio.

### Statistical analyses

All results were presented as means ± standard deviation (SD). Significant values in the groups were assessed using One-Way ANOVA. Statistical analyses were performed using GraphPad and Prism version 7.04 for Windows software (GraphPad Software, San Diego, CA, USA). *P*-values < 0.05 were considered significant.

## Results

### *E*Gb 761 reduces cytosolic calcium levels in a pentylenetetrazole-induced *in vitro* neurotoxicity model in SH-SY5Y cells

**FIGURE 1:**
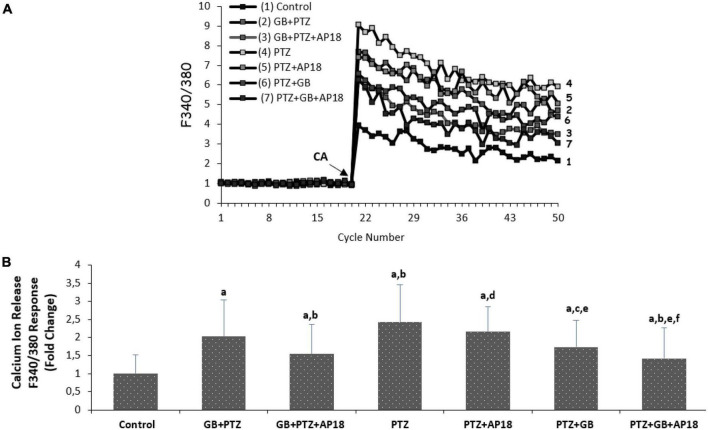
**(A,B)** The effects of pentylenetetrazole (30 mM, for 24 h) and *EGb 761* (25 μg/ml, for 24 h) on cytosolic calcium levels in SH-SY5Y cells. All groups were stimulated on the 20th cycles with 0.1 mM cinnamaldehyde (CA) in the presence of 1.2 mM calcium and calcium-free buffer in the extracellular environment (mean ± SD and *n* = 3). *^a^p* < 0.001 vs. control, *^b^p* < 0.001, and *^c^p* < 0.05 vs. *EGb 761* + PTZ, *^d^p* < 0.001 and *^e^p* < 0.05 vs. PTZ and *^f^p* < 0.001 vs. PTZ + *EGb 761* group.

The effects of *EGb 761* administration on cytosolic calcium levels in pentylenetetrazole-induced SH-SY5Y cells are shown in Figures 1A, B. The TRP ankyrin 1 channel stimulator cinnamaldehyde and blocker AP18 were used to evaluate intracellular Ca^2+^ increase through TRPA1 channels in the cells. It is noteworthy to mention that, for the purpose of distinguishing the function of ion channels in drug assays among groups employing identical chemicals, the cells were first subjected to cinnamaldehyde stimulation, followed by treatment with PTZ and potential drugs, including *EGb 761* or AP18. As a result, a channel stimulator known as Cinnamaldehyde was administered to a particular group, while a combination of a channel inhibitor and a channel stimulator (AP18 + Cinnamaldehyde) was administered to another group.

As shown in Figure 1A, the Ca^2+^ concentration in SH-SY5Y was greater in the *EGb 761* + pentylenetetrazole, pentylenetetrazole, and pentylenetetrazole + *EGb 761* groups compared with the control group (*p* < 0.001, for all). Ca^2+^ levels were lower in the *EGb 761* + pentylenetetrazole and pentylenetetrazole + *EGb 761* groups than in the pentylenetetrazole group (*p* < 0.001 and *p* < 0.05). Cytosolic Ca^2+^ concentrations were also lower in the pentylenetetrazole + *EGb 761* group than in the *EGb 761* + pentylenetetrazole (*p* < 0.05) group. The values were also higher in the *EGb 761* + pentylenetetrazole, pentylenetetrazole and pentylenetetrazole + EGb 761 groups compared with the *EGb 761* + pentylenetetrazole + AP18, pentylenetetrazole + AP18 and pentylenetetrazole + *EGb 761* + AP18 groups (*p* < 0.001 and *p* < 0.05).

### *EGb 761* reduces apoptosis and intracellular reactive oxygene species levels in the pentylenetetrazole-induced *in vitro* neurotoxicity model in the SH-SY5Y cells

**FIGURE 2:**
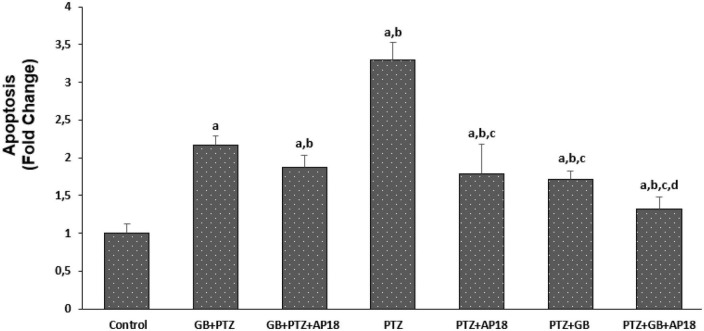
The effect of pentylenetetrazole (PTZ, 30 mM, 24 h) and *EGb 761* (25 μg/ml, 24 h) administration on apoptosis levels in SHSY-5Y cells. Cells were stimulated by cinnamaldehyde (CA, 100 μM for 10 min) but inhibited by AP18 (100 mμ, 30 min) (mean ± SD and *n* = 10). *^a^p* < 0.001 vs. control, *^b^p* < 0.001 vs. *EGb 761* + PTZ, *^c^p* < 0.001 vs. PTZ and *^d^p* < 0.001 vs. PTZ + *EGb 761*.

**FIGURE 3:**
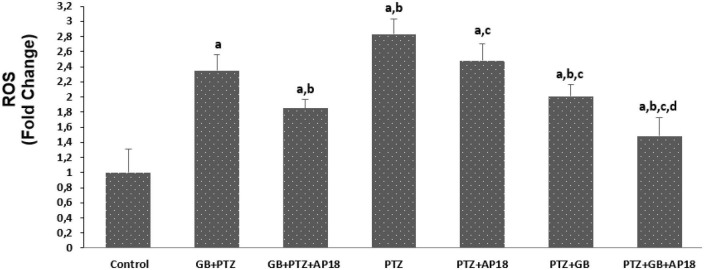
The effect of pentylenetetrazole (PTZ, 30 mM, 24 h) and *EGb 761* (25 μg/ml, 24 h) administration on reactive oxygene levels in the SHSY-5Y cells. Cells were stimulated by cinnamaldehyde (CA, 100 μM for 10 min) but inhibited by AP18 (100 mμ, 30 min) (mean ± SD and *n* = 10). *^a^p* < 0.001 vs. control, *^b^p* < 0.001 vs. *EGb 761* + PTZ, *^c^p* < 0.001 vs. PTZ and *^d^p* < 0.001 vs. PTZ + *EGb 761*.

The effects of *EGb 761* administration on apoptosis and intracellular ROS levels in neuroblastoma cells are shown in Figures 2, 3. Apoptosis and intracellular ROS values were higher in the *EGb 761* + pentylenetetrazole, pentylenetetrazole, and pentylenetetrazole + *EGb 761* groups compared with the control group (*p* < 0.001). These values were also higher in the pentylenetetrazole group than in the *EGb 761* + pentylenetetrazole and pentylenetetrazole + *EGb 761* groups (*p* < 0.001), and higher in the *EGb 761* + pentylenetetrazole group than in the pentylenetetrazole + *EGb 761* group (*p* < 0.001).

The values were also higher in the *EGb 761* + pentylenetetrazole, pentylenetetrazole and pentylenetetrazole + *EGb 761* groups compared with the *EGb 761* + pentylenetetrazole + AP18, pentylenetetrazole + AP18, and pentylenetetrazole + *EGb 761* + AP18 groups (*p* < 0.001 and *p* < 0.05).

### *EGb 761* reduces mitochondrial depolarization, caspase 3, and caspase 9 in the pentylenetetrazole induced *in vitro* neurotoxicity model in SH-SY5Y cells

**FIGURE 4:**
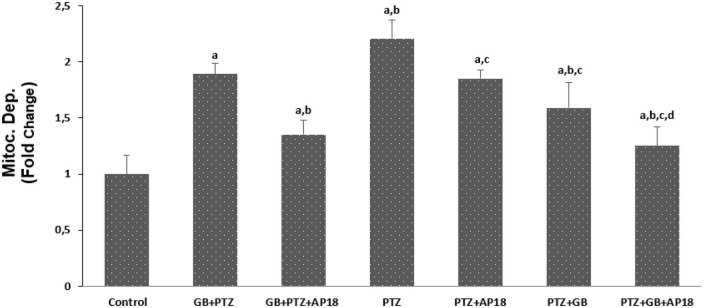
The effects of pentylenetetrazole (PTZ, 30 mM, 24 h) and *EGb 761* (25 μg/ml, 24 h) administration on mitochondrial depolarization levels in SHSY-5Y cells. Cells were stimulated by cinnamaldehyde (CA, 100 μM for 10 min) but inhibited by AP18 (100 mμ, 30 min) (mean ± SD and *n* = 10). *^a^p* < 0.001 vs. control, *^b^p* < 0.001 vs. *EGb 761* + PTZ, *^c^p* < 0.001 vs. PTZ and *^d^p* < 0.001 vs. PTZ + *EGb 761*.

**FIGURE 5:**
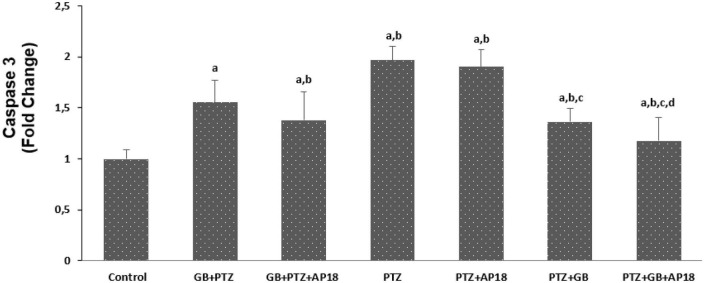
The effects of pentylenetetrazole (PTZ, 30 mM, 24 h) and *EGb 761* (25 μg/ml, 24 h) administration on caspase-3 levels in SHSY-5Y cells. Cells were stimulated by cinnamaldehyde (CA, 100 μM for 10 min) but inhibited by AP18 (100 mμ, 30 min) (mean ± SD and *n* = 10). *^a^p* < 0.001 vs. control, *^b^p* < 0.001 vs. *EGb 761* + PTZ, *^c^p* < 0.001 vs. PTZ and *^d^p* < 0.001 vs. PTZ + *EGb 761*.

**FIGURE 6:**
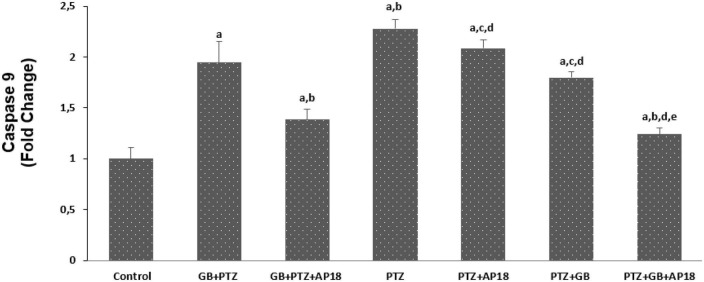
The effects of pentylenetetrazole (PTZ, 30 mM, 24 h) and *EGb 761* (*EGb 761*, 25 μg/ml, 24 h) on caspase-9 levels in SHSY-5Y cells. Cells were stimulated by cinnamaldehyde (CA, 100 μM for 10 min) but inhibited by AP18 (100 mμ, 30 min) (mean ± SD and *n* = 10). *^a^p* < 0.001 vs. control, *^b^p* < 0.001 and *^c^p* < 0.05 vs. *EGb 761* + PTZ, *^d^p* < 0.001 vs. PTZ and *^e^p* < 0.001 vs. PTZ + *EGb 761*.

The effects of *EGb 761* administration on mitochondrial depolarization, caspase 3, and caspase 9 levels in neuroblastoma cells are shown in Figures 4–6. Mitochondrial depolarization, caspase 3, and caspase 9 levels were higher in the *EGb 761* + pentylenetetrazole, pentylenetetrazole and pentylenetetrazole + *EGb 761* groups compared with the control group (*p* < 0.001). Higher values were also observed in the pentylenetetrazole group than in the *EGb 761* + pentylenetetrazole and pentylenetetrazole + *EGb 761* groups (*p* < 0.001). Mitochondrial depolarization and caspase 3 and caspase 9 levels were lower in the *EGb 761* + pentylenetetrazole group than in the pentylenetetrazole + *EGb 761* group (*p* < 0.001). Higher values were also found in the *EGb 761* + pentylenetetrazole, pentylenetetrazole and pentylenetetrazole + *EGb 761* groups than in the *EGb 761* + pentylenetetrazole + AP18, pentylenetetrazole + AP18, and pentylenetetrazole + *EGb 761* + AP18 groups (*p* < 0.001 and *p* < 0.05). However, no significant difference was observed in terms of caspase 3 between the pentylenetetrazole and pentylenetetrazole + AP18 groups.

## Discussion

Our study demonstrated that ROS and calcium levels along with various other apoptotic markers (such as, mitochondrial depolarization and caspase levels) are produced during seizures in the PTZ kindling model of epilepsy that have been effectively reversed with *EGb 761* treatment.

Pentylenetetrazole (PTZ) is a GABA-A receptor antagonist widely used as a seizure inducer by inhibiting the synaptic functions leading to increased neuronal activity. Amongst all animal models of seizure and epilepsy, the pentylenetetrazole-induced seizures are categorized as a model of generalized seizure (vs. partial or focal seizure). In this context, a single systemic injection of PTZ is usually sufficient to elicit seizures, and recovery is rapid. Therefore, the PTZ-kindling model allows for greater regulation of the seizure frequency that is more controllable, effective and reproducible compared to many other chemical kindling models in where the generated epilepsy paradigm is more challenging, and the seizure severity and mortality are uncontrollable (Shimada and Yamagata, 2018). Apoptosis is an important part of the epilepsy process involving numerous cells signaling pathways (Ciftci et al., 2020; Ozansoy et al., 2020; Sever et al., 2022). Mitochondrial destabilization, oxidative stress production, increased cytoplasmic calcium, and elevated caspase-3 and caspase 9 levels are critical apoptosis mediators. This study therefore evaluated the protective effects of *EGb 761* extract in pentylenetetrazole-induced *in vitro* epilepsy conditions. The results showed that *EGb 761* reduced mitochondrial apoptosis, caspase release, and ROS formation, while ameliorating apoptotic cell death and calcium influx in pentylenetetrazole-treated SH-SY5Y cells. During further evaluation of the underlying mechanism, we also observed that additional TRPV1 channel blockade significantly increased the neuroprotective effects of *EGb 761*. Considering the epileptogenic potential of pentylenetetrazole as well as the stabilization of impaired pathophysiological markers such as calcium influx, mitochondrial destabilization implicated in epileptogenesis, our present findings might indirectly support the idea in the previous literature of the role of TRP1 channels in the development of epilepsy including especially drug resistant epilepsy subtypes, as well as the direct protective and anticonvulsant effects of *EGb 761*.

*EGb 761* applications both before and after the pentylenetetrazole incubation period reduced Ca release and restored apoptosis, ROS changes, mitochondrial depolarization and caspase levels. These findings suggest a prominent prophylactic and therapeutic effect of *EGb 761* in the pentylenetetrazole-induced epileptogenesis process. However, despite this difference observed in both *EGb 761* application groups, the addition of AP18, a TRPV channel blocker, significantly diminished the epileptic process without leading to a significant difference between these groups (Group 3 vs. Group 7). This again suggested that TRPV1 channels play a pivotal role in the epileptogenesis process, indicating their well-known role in epilepsy-related brain damage mechanisms, such as excitotoxicity, glutamate release, and neuronal apoptosis. It may also be interesting to investigate whether enhanced thermogenesis exerts an indirect effect via activation of TRPV channels, which is, to the best of our knowledge, an unsolved question.

Our findings contrast with a previous study by Pilija et al. (2010) who reported that that *EGb 761* increased epilepsy-induced neuronal discharge in an animal model. Some studies in this field have examined n-vitro effects, while others have focused on vivo animal studies and human clinical and autopsy reports. There are also a number of clinical data concerning suspected interactions between *EGb 761* extract and antiepileptic drugs, which may be related to different methods of *EGb 761* administration, such as either in plant extract form or the plant itself. Here it is worth mentioning that different products from the same plant as well as the considerable difference between different extracts might be misleading that there is a strong link between epilepsy and *EGb 761* (Kulić et al., 2022). Furthermore, several studies with high quality extracts have indicated that there is no evidence for more frequent incidence of seizures in epileptic and non-epileptic patients. It is also worth mentioning that there are also minor changes that should be discussed. For instance, the non-significance observed between PTZ + AP18 vs. PTZ + *EGb 761* in terms of caspase, calcium and ROS changes suggest a comparable beneficial effect of *EGb 761* with AP18 on Ca levels and oxidative and apoptotic markers in this model. Also, the different caspase responses observed between PTZ + AP18 vs. PTZ + *EGb 761* groups might be explained with the fact (where a significant difference was observed between the caspase 3 levels that was not reflected in caspase 9 levels) that caspase 3 and caspase 9 are involved in different epileptic neuronal apoptosis pathways (Méndez-Armenta et al., 2014). Moreover, the unneglectable difference of Ca, caspase, ROS and mitochondrial depolarization markers between the *EGb 761* + PTZ + AP18 vs. PTZ groups can be explained with the fact that the application of *EGb 761* after PTZ stimulation is more effective compared to its application before inducing the epilepsy process suggesting its therapeutic effect instead of a possible prophylactic effect.

In addition to providing valuable preclinical data on its neuroprotective effects, our findings are also intriguing in terms of *EGb 761*’s clinical application in the elderly population presenting with cognitive impairment/mild dementia but who are also predisposed to developing epilepsy. Several studies have revealed that dementia may also include the degradation of crucial areas implicated in epileptogenesis. Palop et al. (2007) for example, shown that the two illnesses are intimately linked, and that it is difficult to create a clear pathophysiological distinction between these two overlapping conditions. In other words, not only are individuals with dementia disorders, such as Alzheimer’s disease, at a greater risk of acquiring epilepsy, but those with epilepsy are also at a greater risk of developing dementia. This is consistent with a revised definition of epilepsy and dementia in temporal lobe epilepsy patients. Apart from the question of causal linkages between epilepsy and dementia, it is intriguing that *EGb 761* exhibited a neuroprotective effect in the present study, which is linked to TRPVA channel modification. In terms of the recently reported bidirectional association between epilepsy and cognitive impairment, although our present data provide no direct evidence for the anticonvulsant effect of *EGb 761*, our findings strongly exclude an epileptogenic potential of *EGb 761* and send an important message that *EGb 761* exerts a significant neuroprotective effect implying an effect that may involve shared pathophysiological mechanisms responsible for both epilepsy and dementia conditions.

Since we employed *in vitro* experimental conditions with a tight experimental design to produce findings capable of extrapolation to human beings, our results may be more acceptable than some other previously published data. Furthermore, our present findings might be also extrapolated to different epilepsy subtypes due to the fact that the pentylenetetrazole-induced seizures are categorized as a model of generalized seizure and TRCP receptors have been increasingly mentioned in the pathophysiology of temporal epilepsy and drug-resistant epilepsy subtypes (Rocha et al., 2020; Zheng, 2022). Multiple studies have been conducted to develop genetic mouse models of epilepsy and employ electrode implantation techniques, resulting in the successful generation of mice that display spontaneous seizures (Seyfried and Glaser, 1985; Upton and Stratton, 2003; Pitkänen et al., 2006; Löscher, 2011; Loscher, 2017). However, these methods are associated with high costs, technical complexity, and the need for surgical expertise in brain implantation techniques (Pitkänen, 2006). Additionally, they do not provide accurate predictions of seizure occurrence in genetic models, necessitating the use of an additional monitoring system to observe epileptic behavior (Yang and Frankel, 2004). On the other hand, pharmacologically induced seizure induction methods are frequently employed techniques to study the underlying mechanisms of epilepsy pathology (Kandratavicius et al., 2014). Among them, pharmacologically induced seizure induction, particularly through the use of pentylenetetrazol (PTZ), is cost-effective and easy to comprehend and offers notable advantages due to its ability to effectively control the timing and frequency of seizures (Shimada et al., 2018). Thus, this novel method represents a straightforward and uncomplicated approach to eliciting intense seizures and enables to adjust dose-dependent modification of epileptic seizure severity resulting in the utilization of this approach for the evaluation of anti-epileptic medications, and genes associated with epilepsy, including also drug resistant epilepsy subtypes Seyfried and Glaser (1985), Löscher (2002), and Upton and Stratton (2003). Furthermore, this approach has been employed to examine neuronal injury subsequent to epileptic seizures, as the histological alterations observed in the brains of individuals with epilepsy are also evident in the brains of animals chemically induced to experience seizures (Mizoguchi et al., 2011). Therefore, this protocol demonstrates its utility in the efficient generation of animal models for studying epilepsy in humans, particularly in relation to specific subtypes human temporal epilepsy, a region known to play a crucial role in the development of drug resistant epileptogenesis and observed in both in humans and animal types of epilepsy.

The results of this study suggest that *EGb 761*’s potential epileptic qualities may be connected to its impact on enhancing the elimination of antiepileptic medicines rather than a direct convulsant action, implying that it may be safely used in the older population with an elevated seizure risk and even employed in older epileptic patients. The current results align with prior research indicating that extracts of high quality, such as those utilized in this study, do not exhibit indications of epileptogenic potential. Conversely, low quality ginkgo seeds have been associated with a higher incidence of epileptogenic activity, which has been attributed to the presence of a neurotoxic compound 4′-O-methylpyridoxine (Wada et al., 1988) found only in trace amounts in high quality Gingko biloba extracts, such as *EGB 761*. In conclusion, the study findings suggest that stabilization of mitochondria, cytoplasmic caspase and Ca levels, as well as normalized oxidative stress during seizures, may become to represent an additional therapeutic target in epilepsy, and that prescribing *EGb 761* to patients with cognitive impairment and epilepsy may be beneficial, with no side-effects developing. Here, it is worth noting that our findings provide important clue of the clinical application of EGB 761 in elderly population with a potential cognitive impairment. Although our study provides valuable data, we are not able to provide additional data regarding detailed signal transduction upstream or downstream pathways of TRPA, with additional experimental set ups (such as PTZ plus AP18 alone; and with AP18 alone; and with GB alone) due to economic reasons. We are aware that this makes it difficult to estimate whether and to what extent the observed effects are indeed mediated via the TRP ankyrin 1 channel which can be considered as a limitation. It is also noteworthy to mention that the assessment of the calcium signal is not viable in the absence of cinnamaldehyde and that PTZ does not function as a receptor agonist in directly stimulating the TRPA1 channel. Instead, PTZ induces the activation of the TRPA1 channel by increasing intracellular levels of reactive oxygen species (ROS), thereby causing oxidative stress.

In conclusion, it is of the utmost importance to notice that TRPA1 channels exhibit selective activation in response to oxidative stress and function as stimulatory agents rather than direct agonists. Based on the established framework, it is justifiable to suggest that the disparity observed between the two groups when subjected to AP18 + CA stimulation compared to solely CA may yield significant insights into the efficacy of PTZ’s indirect influence on the channel.

Nevertheless, there has been no other study, to our knowledge, that has examined the combined effects of *EGb 761* and pentylenetetrazole on TRP channels while also measuring their level of activation.

In conclusion, despite the aforementioned limitations arising also from the lack of a patch clamp system, which hindered our ability to measure ion channel activation at detailed individual cell level, we successfully developed a basic mechanistic framework elucidating the pathophysiological significance of fundamental ion mechanisms in a pentylenetetrazole treated neuroblastoma cell line. One of the primary rationales behind the design of our experiments was to investigate the impact of *EGb 761* in a more controlled model of pentylenetetrazole-induced generalized epilepsy. This model holds particular clinical significance as it closely resembles drug-resistant epilepsy, a condition that represents significant therapeutic challenges for epileptologists. Our findings indicate that *EGb 761* exhibited a reduction in Ca release and subsequent restoration of apoptosis, ROS changes, mitochondrial depolarization and caspase levels, devoid of any adverse effects, thereby supporting its favorable efficacy and safety profile as previously indicated by multiple *in vivo* and *in vitro* models. Further investigation is required to explore the therapeutic potential of *EGb 761* in individuals with cognitive impairments who are susceptible to developing epilepsy. Additionally, conducting *in vivo* studies to validate this protective effect would provide substantial evidence supporting its therapeutic application.

As a conclusion, beyond demonstrating that the restoring of apoptotic and oxidative process may result with significant prophylactic and therapeutic effect, our current results suggest that EGB 761 may be a novel therapeutic target in epilepsy with its potential antiapoptotic, anticonvulsant, and antioxidant features making it a potential therapeutic candidate in old patients with cognitive impairment, with no stimulating side-effects developing.

## Data availability statement

The original contributions presented in this study are included in the article/supplementary material, further inquiries can be directed to the corresponding authors.

## Ethics statement

The studies involving humans were approved by the Medipol Institutional Ethical Board. The studies were conducted in accordance with the local legislation and institutional requirements. Written informed consent for participation in this study was provided by the participants’ legal guardians/next of kin. Written informed consent was obtained from the individual(s), and minor(s)’ legal guardian/next of kin, for the publication of any potentially identifiable images or data included in this article.

## Author contributions

BY and AM: editing supervision writing. IO and AO: experiments. OA and HV: designing, analyzing the data. All authors contributed to the article and approved the submitted version.
